# microRNA-1246-containing extracellular vesicles from acute myeloid leukemia cells promote the survival of leukemia stem cells via the LRIG1-meditated STAT3 pathway

**DOI:** 10.18632/aging.202893

**Published:** 2021-04-23

**Authors:** Lijuan Chen, Zhi Guo, Yongming Zhou, Jian Ni, Jianhua Zhu, Xu Fan, Xuexing Chen, Yiling Liu, Ziping Li, Hao Zhou

**Affiliations:** 1 Department of Obstetrics and Gynecology, Union Hospital, Tongji Medical College, Huazhong University of Science and Technology, Wuhan 430022, China; 2 Department of Hematology and Oncology, National Cancer Center/National Clinical Research Cancer for Cancer/Cancer Hospital and Shenzhen Hospital, Chinese Academy of Medical Sciences and Peking Union Medical College, Shenzhen 518116, China; 3 Department of Hematology, The Affiliated Tianyou Hospital, Wuhan University of Science and Technology, Wuhan 430064, China; 4 Department of Oncology Clinical Pharmacy, Brigham and Women's Hospital, Harvard Medical School, Boston, MA 02115, USA; 5 Laboratory of Clinical Immunology, Wuhan No. 1 Hospital, Tongji Medical College, Huazhong University of Science and Technology, Wuhan 430022, China; 6 Liver Research Center, Beijing Friendship Hospital, Capital Medical University, Beijing Key Laboratory of Translational Medicine in Liver Cirrhosis and National Clinical Research Center for Digestive Diseases, Beijing 100050, China; 7 Institute of Hematology, Union Hospital, Tongji Medical College, Huazhong University of Science and Technology, Wuhan 430022, China

**Keywords:** acute myeloid leukemia, leukemia stem cells, extracellular vesicle, microRNA-1246, LRIG1

## Abstract

Cancer cells-secreted extracellular vesicles (EVs) have emerged as important mediators of intercellular communication in local and distant microenvironment. Our initial GEO database analysis identified the presence of differentially-expressed microRNA-1246 (miR-1246) in acute myeloid leukemia (AML) cell-derived EVs. Consequently, the current study set out to investigate the role of AML-derived EVs-packaged miR-1246 in leukemia stem cells (LSCs) bioactivities. The predicted binding between miR-1246 and LRIG1 was verified using dual luciferase reporter assay. Then, gain- and loss-of-function assays were performed in LSCs, where LSCs were co-cultured with AML cell-derived EVs to characterize the effects of miR-1246-containing EVs, miR-1246, LRIG1 and STAT3 pathway in LSCs. Our findings revealed, in AML cell-derived EVs, miR-1246 was highly-expressed and directly-targeted LRIG1 to activate the STAT3 pathway. MiR-1246 inhibitor or EV-encapsulated miR-1246 inhibitor was found to suppress the viability and colony formation abilities but promoted the apoptosis and differentiation of LSCs through inactivation of STAT3 pathway by up-regulating LRIG1. In addition, the inhibitory effects of AML cell-derived EVs carrying miR-1246 inhibitor on LSCs were substantiated by *in vivo* experiments. Collectively, our findings reveal that the repression of AML cell-derived EVs containing miR-1246 inhibitor alters the survival of LSCs by inactivating the LRIG1-mediated STAT3 pathway.

## INTRODUCTION

Acute myeloid leukemia (AML) is a heterogeneous disease comprising of numerous genotypes, phenotypes, and epigenetic characteristics, which ultimately dictate the terms of anti-leukemia treatment and potential leukemic relapse, the primary cause of treatment failure [1]. In spite of its heterogeneity, AML does exhibit some common features such as the avoidance of programmed cell death, and resistance to cytotoxic stimuli [2]. A rare population of AML cells, the self-renewable and quiescent leukemia stem cells (LSCs), has been reported to account for the occurrence of cytotoxic resistance in AML. In order to effectively eradicate LSCs and thus treat AML, allogeneic hematopoietic cell transplantation has been developed and successfully implemented in the treatment of AML over the last few decades. Furthermore, new immunotherapies such as immune checkpoint inhibitors, chimeric antigen receptor T cells and, bispecific antibodies, are being increasingly tested and applied in current AML interventions [3]. Although the recent advent of newly launched therapies appears promising, except for acute promyelocytic leukemia, half of AML cases still remain incurable to date. The detailed and precise mechanisms associated with AML development and progression are still obscure, hampering the establishment of better strategies for the elimination of LSCs and, ultimately, for the cure for AML.

Adult bone marrow is the major site of hematopoiesis and as a result, provides a hotspot for the development of certain malignant hematopoietic diseases such as AML. In a physiological context, interactions between the bone marrow microenvironment and hematopoietic stem cells maintain the delicate, but essential balance between proliferation, differentiation and homeostasis of the stem cell compartment. However, when it comes to malignancies, AML cells infiltrate the bone marrow, and then interfere with the normal hematopoietic stem cell microenvironment homeostasis [4]. Emerging evidence on the GSE55025 dataset has suggested that AML extracellular vesicle (EV)-derived microRNAs (miRNAs) could target c-MYB and be delivered into benign hematopoietic stem cells and progenitor cells, which could cause the loss of hematopoietic function in benign cells [5]. Additionally, our previous study demonstrated that malignant hematopoietic stem cells shedding off EVs comprised of intracellular pro-senescence signals and enhanced chemoresistance [6]. All in all, these data and findings implored us to investigate the role of potential miRNAs associated with AML and AML cell-secreted EVs.

As a group of small lipid bilayer-enclosed particles with a diameter of 30 - 140 nm, EVs originate from endosomes and can be secreted by most of cells [7]. EVs typically carry messenger RNAs (mRNAs), proteins, and miRNAs between cells [8]. Interestingly, regulatory miRNAs are known to widely participate in gene translation and transcription, as well as physiological and pathological processes, such as inflammation, ontogenesis and angiogenesis [9]. In addition, miRNAs have been shown to play a crucial regulatory role in AML development by modulating processes like cell proliferation, differentiation, and survival [10]. Furthermore, miR-126 was found to promote the leukemogenesis of LSCs [11]. Our recently published also study showed that miR-34c-5p accelerates the senescence of LSCs [6]. Additionally, miR-1246 has also been shown to enhance the aggressiveness of liver cancer stem cells in terms of self-renewal, drug resistance, tumorigenesis, and metastasis [12]. Down-regulated miR-1246 has also been shown to restrain hepatocellular carcinoma cell migration and invasion [13]. Meanwhile, the human leucine rich repeats and immunoglobulin like domains 1 (LRIG1), belongs to the LRIG family of trans-membrane leucine rich proteins, and is a tumor suppressor which negatively regulates receptor tyrosine kinase signaling [14]. LRIG1 has been reported to be a haploinsufficient inhibitor of platelet-derived growth factor-induced glioma [15]. Moreover, our preliminary research using predictive tools revealed the presence of a binding site for microRNA-1246 on the LRIG1 gene. In the meantime, LRIG1 gene could regulate the STAT3-dependent inflammatory pathway [16]. Hence, we hypothesized that by targeting LRIG1, AML cells secrete miR-1246 containing EVs and facilitate the survival of LSCs. In the current study, we designed a series of *in vivo* and *in vitro* experiments to test our hypothesis, in the hope of finding modalities to improve the quality of life of patients plagued by AML.

## RESULTS

### miR-1246 is over-expressed in AML cell-secreted EVs

**Figure 1:**
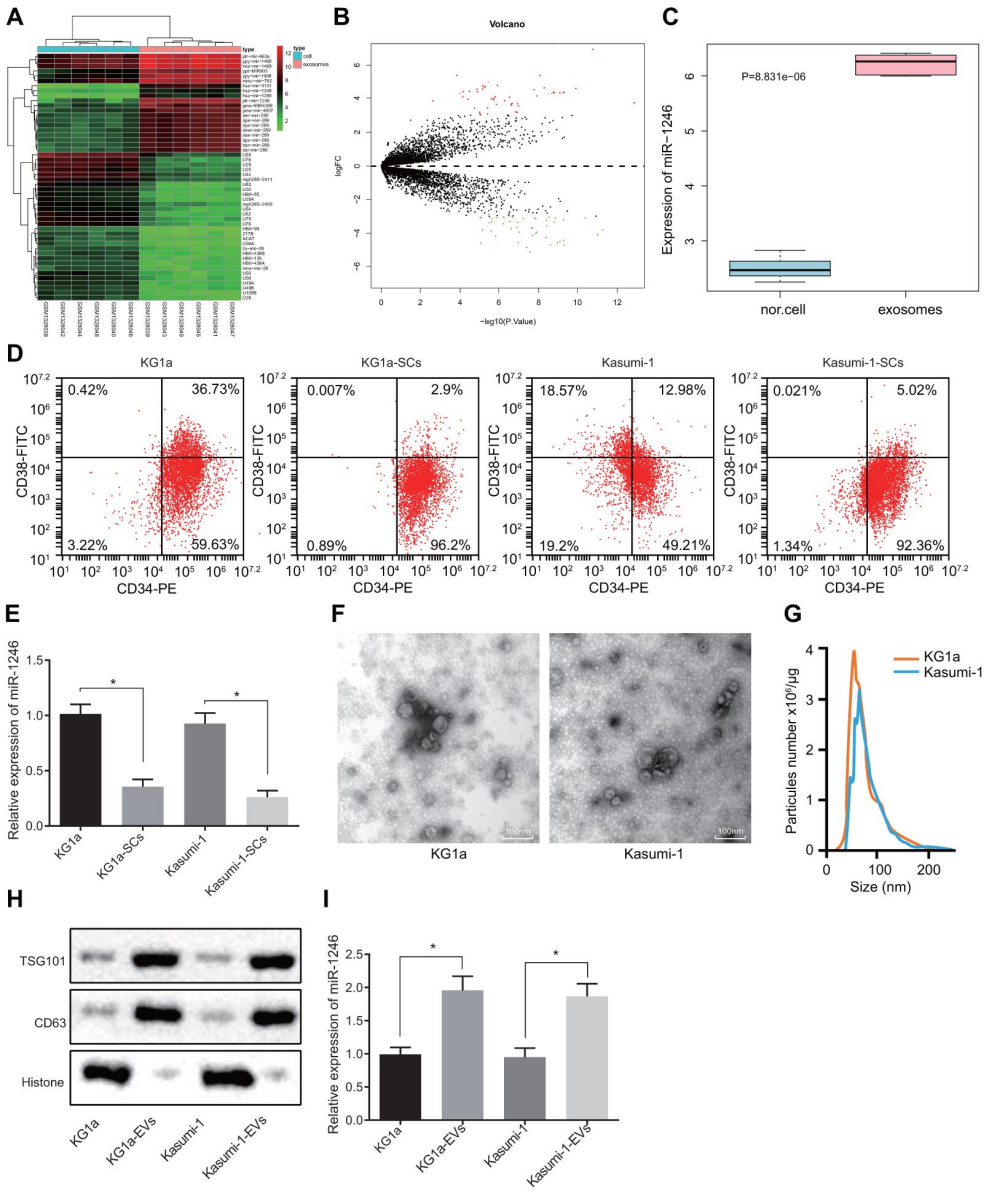
**High levels of miR-1246 expression are identified in AML cell-derived EVs.** (**A**) Heat map of differentially expressed miRNAs from the GSE55025 profile. (**B**) Volcanic map of differentially expressed miRNAs in GSE55025. (**C**) Boxplot of miR-1246 in expression profile GSE55025. The left box refers to expression in normal cell-derived EVs, and the right box represents the expression in AML cell-derived EVs. (**D**) AML cell lines labeled with CD34-PE and CD38-FITC antibodies. The subpopulation of CD34^+^CD38^−^cells (i.e., LSCs) was analyzed by flow cytometry. (**E**) Expression of miR-1246 in AML cell lines (KG1a and Kasumi-1) as well as LSCs detected by RT-qPCR. (**F**) Morphology of AML cell-derived EVs observed by TEM. (**G**) EV concentration and particle size measured by nanoparticle tracking analysis. (**H**) Expression of EV specific markers CD63 and TSG101 assessed by western blot analysis. (**I**) Expression of miR-1246 in AML cell lines (KG1a and Kasumi-1) and in corresponding EVs as detected by RT-qPCR. * *p* < 0.05 *vs.* KG1a or Kasumi-1 cells. Data in the figures are all measurement data, expressed as mean ± standard deviation. Independent sample *t*-test was applied for comparison between two groups. The experiments were repeated in triplicate.

Firstly, differential expression analyses were performed on the AML cell-derived EV-associated RNA expression profile GSE55025 obtained from the GEO database to identify the miRNAs that were significantly differentially-expressed in AML cell-derived EVs, with normal cell-derived EVs serving as the control. Based on the analysis of the expression profile GSE55025, 98 differentially-expressed miRNAs were screened in AML cell-derived EVs, including 53 highly-expressed miRNAs and 45 poorly-expressed miRNAs. The volcanic map and heat map of miRNAs with different expressions (Figure 1A, 1B) were subsequently plotted using the R language. Among these differentially expressed miRNAs, miR-1246 exhibited the greatest significant change in expression (Supplementary Table 1). In addition, the expression data for miR-1246 from each sample were extracted from the GSE55025 profile, and the resulting expression boxplot demonstrated that miR-1246 was predominately expressed in AML cell-derived EVs (Figure 1C). Therefore, we hypothesized that miR-1246 might play a role in leukemia progression. Moreover, miR-1246 has been previously shown to promote the proliferation of leukemia cells [17]. Mounting evidence also suggests that LSCs play a critical role in the pathogenesis and maintenance of AML, which contributes to the likelihood of cancer recurrence and drug resistance [18]. However, studies on the effects of AML-derived EVs on LSCs are scarce. To improve on this, we adopted magnetic bead sorting to separate LSCs from AML cell lines (KG1a and Kasumi-1) to investigate whether AML cell-derived EVs affect LSCs and their biological functions. The ratio of LSCs in the AML cell lines (KG1a and Kasumi-1) was calculated in order to determine whether the LSCs were successfully sorted; CD34-PE and CD38-FITC antibodies were used to stain AML cell lines and LSCs (KG1a-stem cells [SCs] and Kasumi-1-SCs) separated by magnetic beads [19, 20], followed by identification of LSCs using flow cytometry. As shown in Figure 1D, over 95% of cells were CD34^+^CD38^−^, and thus regarded as LSCs after magnetic bead sorting. Furthermore, the expression patterns of miR-1246 in AML cell lines (KG1a and Kasumi-1) and LSCs (KG1a-SCs and Kasumi-1-SCs) were determined, which revealed that the expression of miR-1246 in LSCs was significantly reduced compared to AML cell lines (Figure 1E). By observing the EVs secreted by AML cells, we found that the size of EVs was between 50-150 nm, with EV specific marker proteins being aberrantly expressed (Figure 1F–1H). Subsequently, miR-1246 expression patterns were detected in the EVs, with the results showing that miR-1246 expression in AML cell-derived EVs was significantly higher relative to AML cell lines, which was consistent with the expression profile results (Figure 1I). These results suggested that miR-1246 was highly-expressed in AML cell-derived EVs and poorly-expressed in LSCs.

### AML cell-derived EVs enhanced LSCs survival

**Figure 2:**
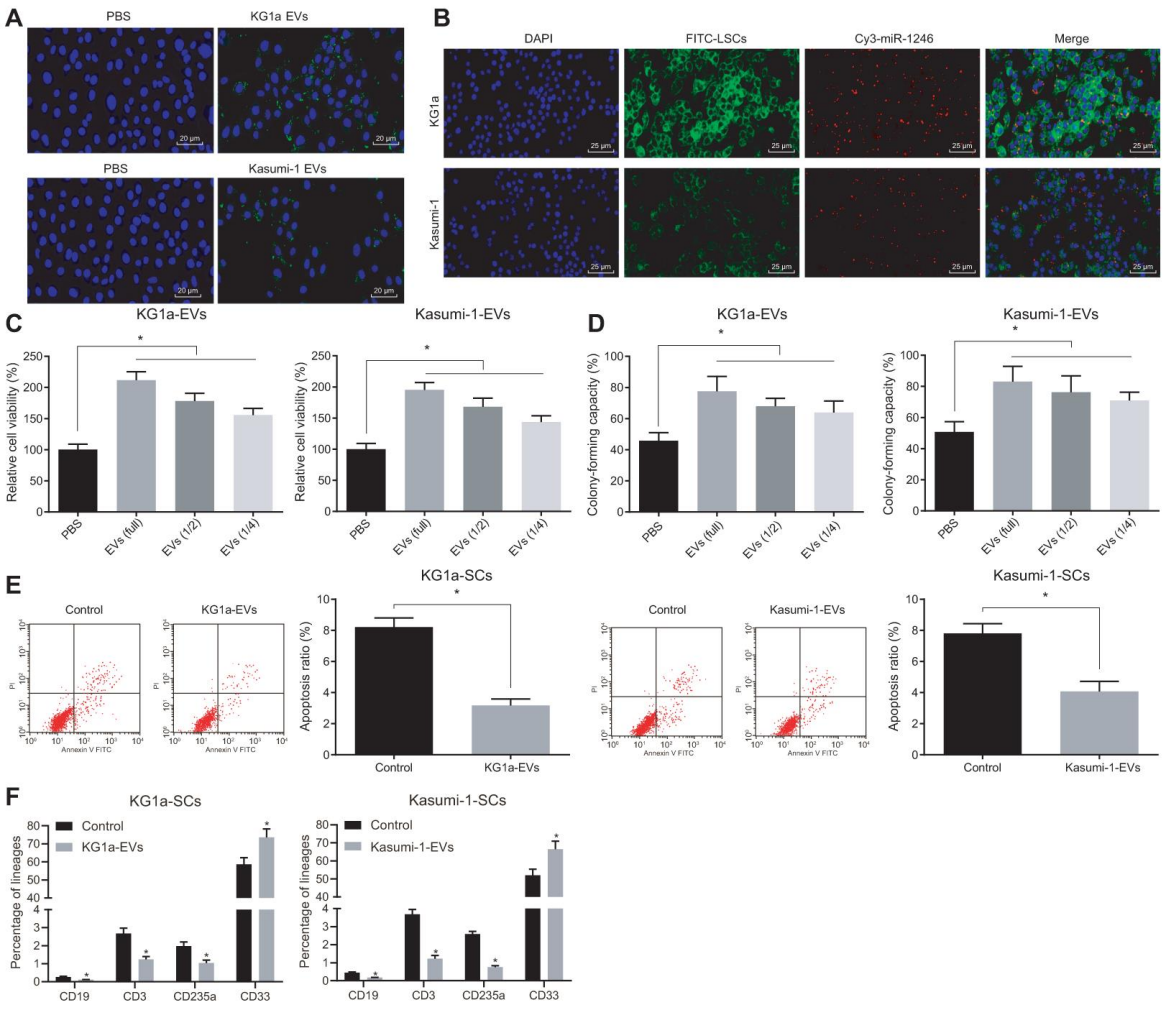
**AML cell-derived EVs elevated the viability and colony formation and reduced the apoptosis and differentiation of LSCs.** (**A**) Absorption of CFSE-labeled AML cell-derived EVs by LSCs observed under a fluorescence microscope (CFSE: green, DAPI: blue; × 500). (**B**) Fluorescence microscopic observation of Cy3 labeled AML cell-derived EVs co-cultured with LSCs (Cy3-miR-1246: red, DAPI: blue, FITC-LSCs: green; × 400). (**C**) Viability of LSCs after exposure to AML cell-derived EVs evaluated by MTS assay. (**D**) Effects of AML cell-derived EVs on the colony formation capacity of LSCs identified by colony formation assay. (**E**) Effects of AML cell-derived EVs on LSCs apoptosis assessed by flow cytometry. (**F**) Changes in differentiation of LSCs after treatment with AML cell-derived EVs detected by differentiation assay. * *p* < 0.05 *vs.* LSCs treated with PBS. Data in the figures are all measurement data, expressed as mean ± standard deviation. Independent sample *t*-test was applied for comparison between two groups. Data among multiple groups were compared by one-way ANOVA. The experiments were repeated in triplicate.

Based on the initial results above, we further conjectured that AML cell-derived EVs could intracellularly enter LSCs to alter the *in vitro* biological functions and differentiation of LSCs, ultimately affecting leukemia progression. After CFSE-labeled AML cell-derived EVs were co-cultured with LSCs for 4 h, the endocytosis of EVs by LSCs was observed under a fluorescence microscope, with the results demonstrating that EVs were internalized by LSCs, indicating that AML cell secreted EVs could target LSCs (Figure 2A). In addition, the red Cy3 fluorescence of LSCs was observed under a fluorescence microscope, indicating that miR-1246 was transferred to LSCs after AML cells were co-cultured with LSCs (Figure 2B). Also, results of MTS and colony assays (Figure 2C, 2D) demonstrated that the cell viability and colony formation abilities of LSCs were positively-correlated to AML cell-derived EVs in a concentration dependent manner. Flow cytometry further revealed that treatment with AML cell-derived EVs significantly reduced the apoptosis of LSCs (Figure 2E). The change in the differentiation ability of LSCs after exposure to AML cell-derived EVs was also assessed, which depicted that the differentiation ability of LSCs was negatively-correlated with the concentration of EVs, evidenced by decreased CD19, CD3 and CD235a positive cells and increased CD33 positive cells (Figure 2F). Taken together, the above results suggested that AML cell-derived EVs promoted the cell viability and colony formation ability of LSCs, but inhibited their apoptosis and differentiation.

### MiR-1246-containing EVs from AML cells targeted LSCs to promote cell survival

**Figure 3:**
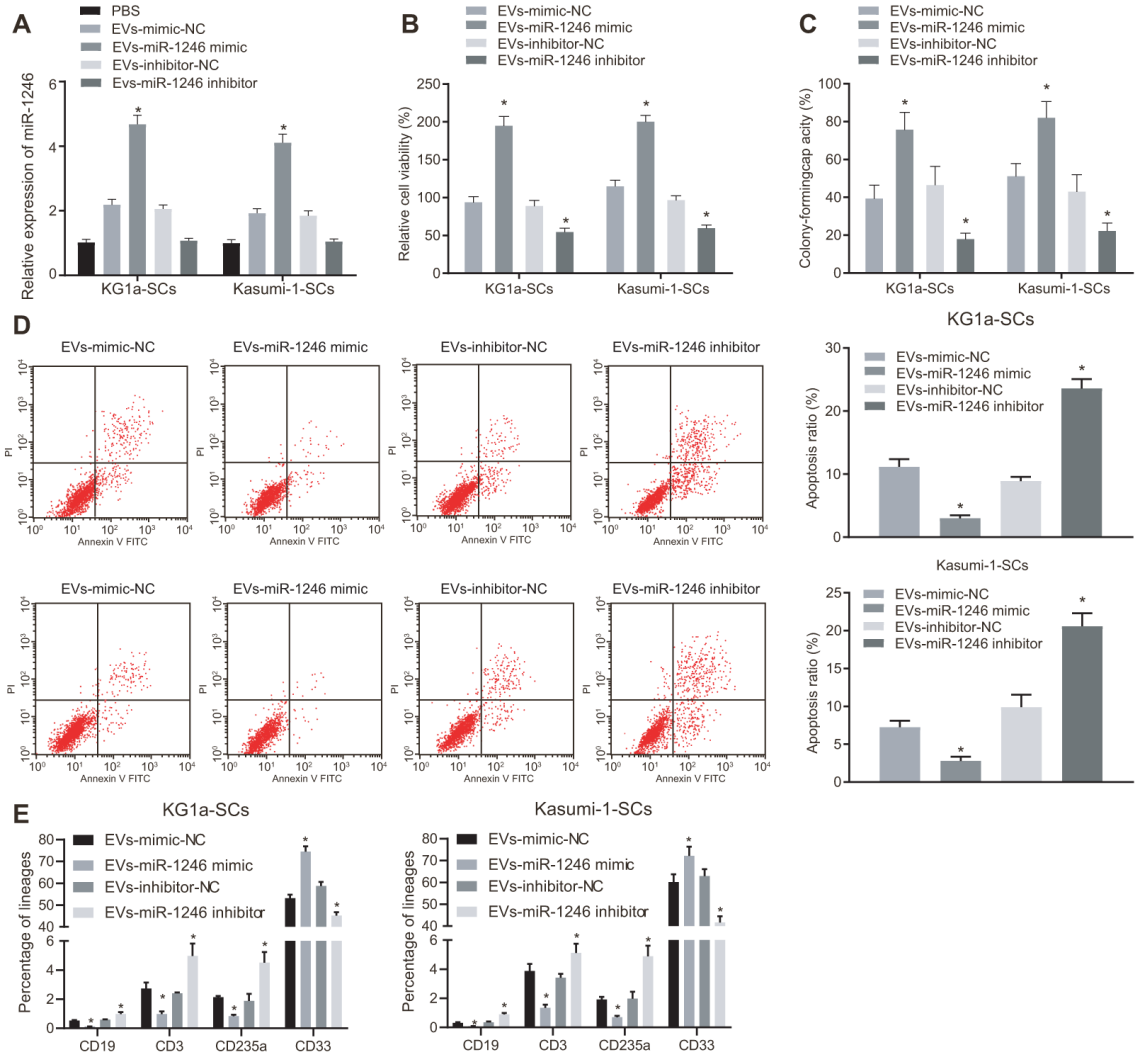
**MiR-1246-containing EVs from AML cells elevated LSCs survival via the regulation of LSCs.** LSCs were co-cultured with PBS, EVs-mimic-NC, EVs-miR-1246 mimic, EVs-inhibitor-NC or EVs-miR-1246 inhibitor. (**A**) Expression of miR-1246 in LSCs detected by RT-qPCR. (**B**) Viability of LSCs assessed by MTS assay. (**C**) Colony formation ability of LSCs tested by colony formation assay. (**D**) Apoptosis of LSCs evaluated by flow cytometry. (**E**) differentiation of LSCs identified by differentiation assay. * *p* < 0.05 *vs.* LSCs co-cultured with EVs-mimic-NC or EVs-inhibitor-NC. Data in the figures are all measurement data, expressed as mean ± standard deviation. Data among multiple groups were analyzed by one-way ANOVA. The experiments were repeated in triplicate.

We next verified whether AML cell-derived EVs containing miR-1246 influenced the biological characteristics and differentiation potential of LSCs *in vitro*. EVs were isolated from AML cells with silenced or over-expressed miR-1246, and co-cultured with LSCs. Then, the role of miR-1246 expression in LSCs was evaluated. MiR-1246 expression was found to be significantly increased in LSCs co-cultured with EVs from AML cells with miR-1246 mimic (i.e. EVs-miR-1246 mimic), while being reduced in LSCs co-cultured with EVs-miR-1246 inhibitor (Figure 3A). In addition, the following assays were performed to better ascertain the effects of each EV type delivered: MTS, cell colony formation, flow cytometry and, differentiation. Cell viability and colony formation abilities were found to be notably elevated, whereas cell apoptosis and differentiation were lowered in LSCs co-cultured with EVs-miR-1246 mimic, which was opposite in LSCs co-cultured with EVs-miR-1246 inhibitor (Figure 3B–3E). To sum up, these findings indicated that miR-1246 carried by AML cell-derived EVs contributed to enhanced LSCs survival by interacting with LSCs.

### AML cell-derived EVs deliver miR-1246 to target and restrain LRIG1 in LSCs

**Figure 4:**
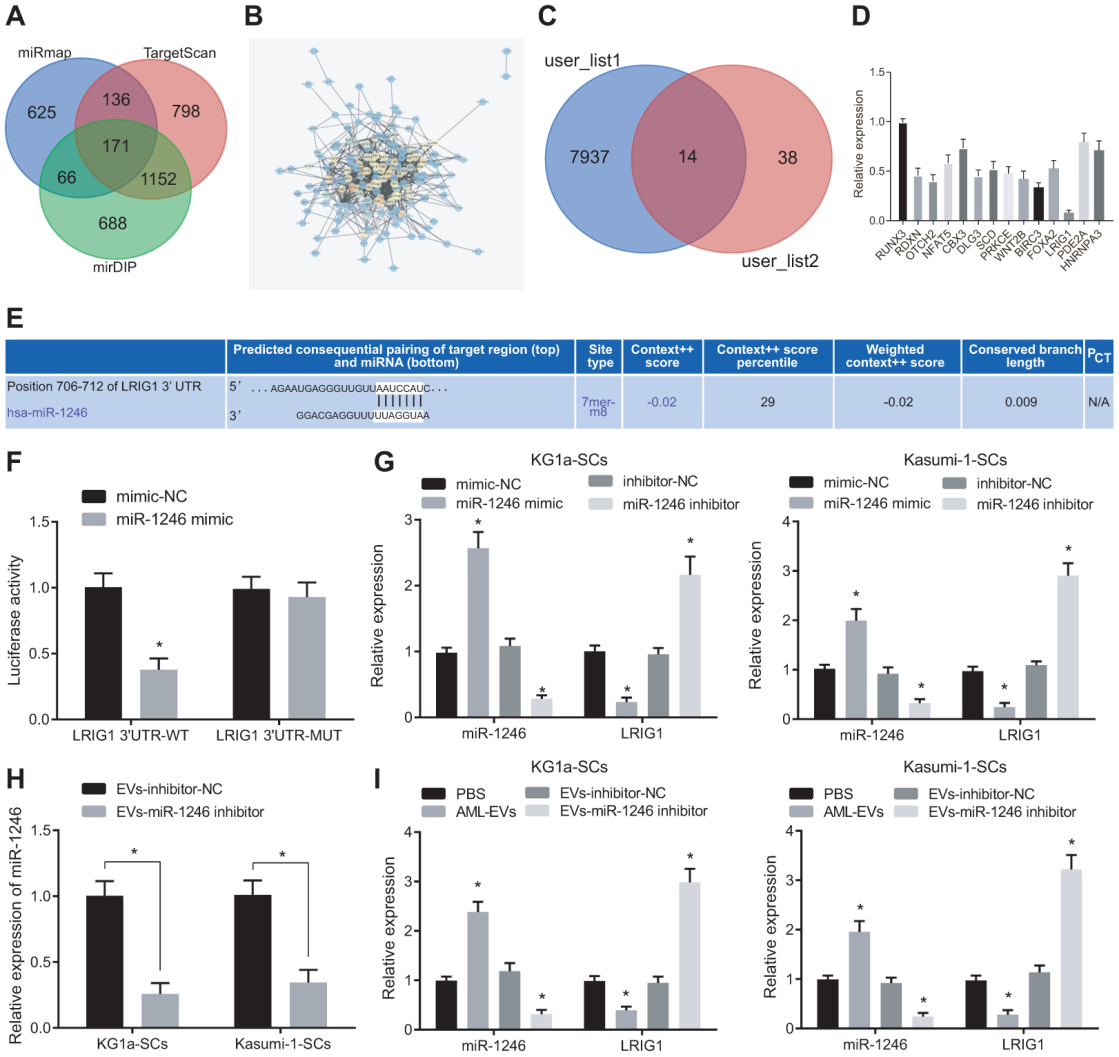
**AML cell-derived EVs containing miR-1246 target LRIG1 in LSCs.** (**A**) Venn diagram of target gene retrieved from databases of mirDIP, TargetscanHuma and miRmap. (**B**) PPI analysis of 171 intersected target genes. (**C**) Venn diagram of 52 genes with junction points ≥ 10 in PPI analysis and low-expressed genes in leukemia from the TCGA database. (**D**) Quantitative analysis of 14 screened genes. (**E**) The binding site of miR-1246 to LRIG1 according to the Targetscan Huma database. (**F**) The binding relationship between miR-1246 and LRIG1 was verified by dual luciferase reporter gene assay. (**G**) miR-1246 and LRIG1 expression in LSCs after transfection with miR-1246 mimic or miR-1246 inhibitor assessed by RT-qPCR. (**H**) Expression of miR-1246 in AML cell secreted EVs with miR-1246 inhibition measured by RT-qPCR. (**I**) Expression of miR-1246 and LRIG1 in LSCs after co-culture with AML cell-derived EVs detected by RT-qPCR. * *p* < 0.05 *vs.* LSCs co-cultured with PBS or EVs-inhibitor-NC. Data in the figures are all measurement data, expressed as mean ± standard deviation. Independent sample *t*-test was applied for comparison between two groups. Data among multiple groups were analyzed by one-way ANOVA. The experiments were repeated in triplicate.

Furthermore, the putative targets of miR-1246 were screened. A total of 2077 target genes with a comprehensive mirDIP score greater than 0.15, 2261 target genes with TargetscanHuma weight comprehensive score greater than 0.02 and the top 1000 target genes in miRmap were screened, wherein 171 target genes were found to be at the intersection according to Venn diagram analysis (Figure 4A). Protein-protein interaction (PPI) analysis was then performed on these 171 genes to obtain 52 genes with more than 10 junction points (Figure 4B). In addition, 14 genes were obtained from Venn diagram analysis of the down-regulated genes of leukemia in TCGA database analyzed by online analysis website GEPIA2 and these 52 genes with junction points more than 10 (Figure 4C). LRIG1 expression patterns were found to be more significantly downregulated in KG1a cells through quantitative analysis (Figure 4D).

Moreover, it has been reported that LRIG1 expression is downregulated in leukemia [21]. Thus, we chose to focus on LRIG1 as a potential target of miR-1246. Most importantly, TargetscanHuma (Figure 4E) predicted the presence of a specific binding site between miR-1246 and LRIG1, which consistently suggested that LRIG1 may be a target of miR-1246. Hence, we speculated that miR-1246 could inhibit the biological functions and differentiation of LSCs *in vitro* by targeting LRIG1. To this end, we verified the interaction between miR-1246 and LRIG1 using dual luciferase reporter gene assay, which indicated that the luciferase activity of LRIG1-3'-UTR^W^ was significantly decreased by miR-1246 mimic. In contrast to the co-transfection of mimic-NC + LRIG1-3'-UTR^M^, the fluorescence activity of LSCs was not significantly altered in response to co-transfection with miR-1246 mimic + LRIG1-3'-UTR^M^ (Figure 4F).

In order to further verify whether miR-1246 could regulate the LRIG1 expression in LSCs, the expression patterns of miR-1246 and LRIG1 were detected in LSCs after transfections with miR-1246 mimic and miR-1246 inhibitor. The results were opposite in response to the transfection of miR-1246 inhibitor (Figure 4G). Subsequently, AML cell lines were silenced with miR-1246 and their secreted EVs were evaluated. It was found that miR-1246 expression was significantly decreased in EVs with EVs-miR-1246 inhibitor (Figure 4H). In addition, isolated EVs were co-cultured with LSCs to detect the expression pattens of miR-1246 and LRIG1 in LSCs, and the results showed that miR-1246 expression was significantly up-regulated, while LRIG1 expression was decreased in LSCs following co-culture with AML-EVs. Meanwhile, miR-1246 expression was reduced, while LRIG1 expression was increased in LSCs in response to co-culture with EVs-miR-1246 inhibitor (Figure 4I). The above results indicated that miR-1246 carried AML cell-derived EVs could target and inhibit LRIG1 expression in LSCs.

### MiR-1246 down-regulates LRIG1 enhancing the survival of LSCs and repressing their differentiation

**Figure 5:**
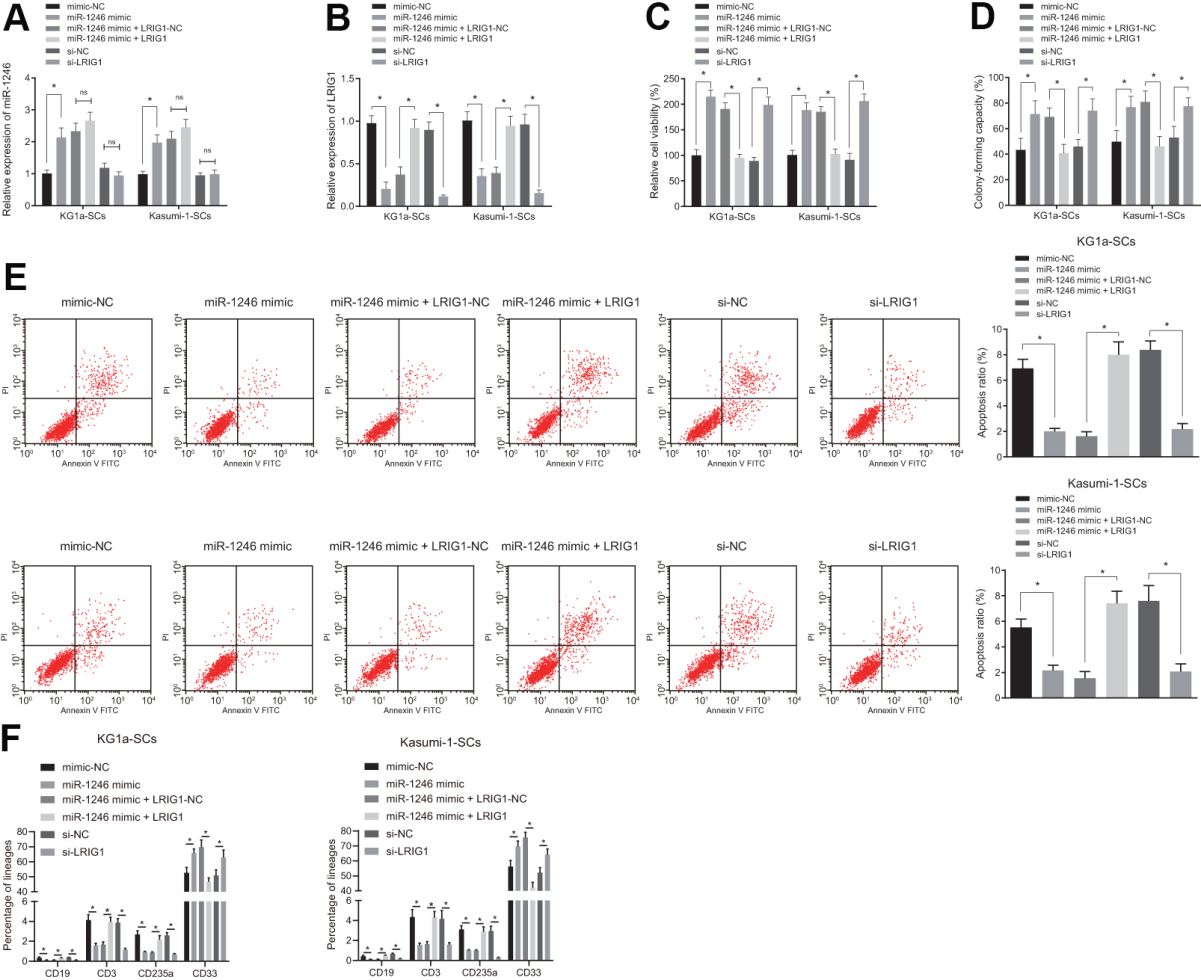
**miR-1246 facilitates the cell survival and impedes differentiation of LSCs by targeting LRIG1.** LSCs were transfected with mimic-NC, miR-1246 mimic, miR-1246 mimic + LRIG1-NC, miR-1246 mimic + LRIG1, si-NC and si-LRIG1. (**A**, **B**) Expression of miR-1246 and LRIG1 in LSCs detected by RT-qPCR after transfection. (**C**) LSCs viability detected by MTS assay. (**D**) Colony formation of LSCs evaluated by colony formation assay. (**E**) LSCs apoptosis identified by flow cytometry. (**F**) Differentiation of LSCs determined by differentiation assay. * *p* < 0.05 *vs.* LSCs transfected with mimic-NC, miR-1246-mimic + LRIG1-NC or si-NC. Data in the figures are all measurement data, expressed as mean ± standard deviation. Data among multiple groups were analyzed by one-way ANOVA. The experiments were repeated in triplicate.

To verify whether AML cell-derived EVs inhibited LRIG1 by delivering miR-1246, thereby affecting the biological characteristics and differentiation of LSCs *in vitro*, over-expressing or silencing LRIG1 was performed as well as miR-1246 mimic was introduced to LSCs for further verification. Firstly, the expression patterns of miR-1246 and LRIG1 in LSCs were detected using RT-qPCR, which showed that the expression of miR-1246 was significantly increased, while that of LRIG1 was significantly decreased in LSCs after transfection with miR-1246 mimic. In contrast to the co-transfection of miR-1246 mimic + LRIG1-NC, the expression of miR-1246 did not differ greatly, while the expression of LRIG1 was found to be significantly up-regulated in LSCs in response to transfection with miR-1246 mimic + LRIG1. Moreover, miR-1246 expression was similar, while LRIG1 expression decreased in LSCs following transfection with si-LRIG1 (Figure 5A, 5B).

In addition, it was observed that relative to mimic-NC, the viability and colony formation were increased, while apoptosis and differentiation were reduced in LSCs in response to transfection with miR-1246 mimic. Meanwhile, co-transfection with miR-1246 mimic + LRIG1 resulted in reduced viability and colony formation, and elevated apoptosis and differentiation in LSCs, compared to co-transfection with miR-1246 mimic + LRIG1-NC. Cell viability and colony formation abilities of LSCs were up-regulated, while cell apoptosis and differentiation were inhibited in response to transfection with si-LRIG1, compared to si- NC (Figure 5C–5F). These results suggested that miR-1246 could promote the viability and suppress apoptosis and differentiation of LSCs by targeting LRIG1.

### MiR-1246 targets LRIG1 to activate the STAT3 pathway, thereby promoting survival and restraining differentiation of LSCs

**Figure 6:**
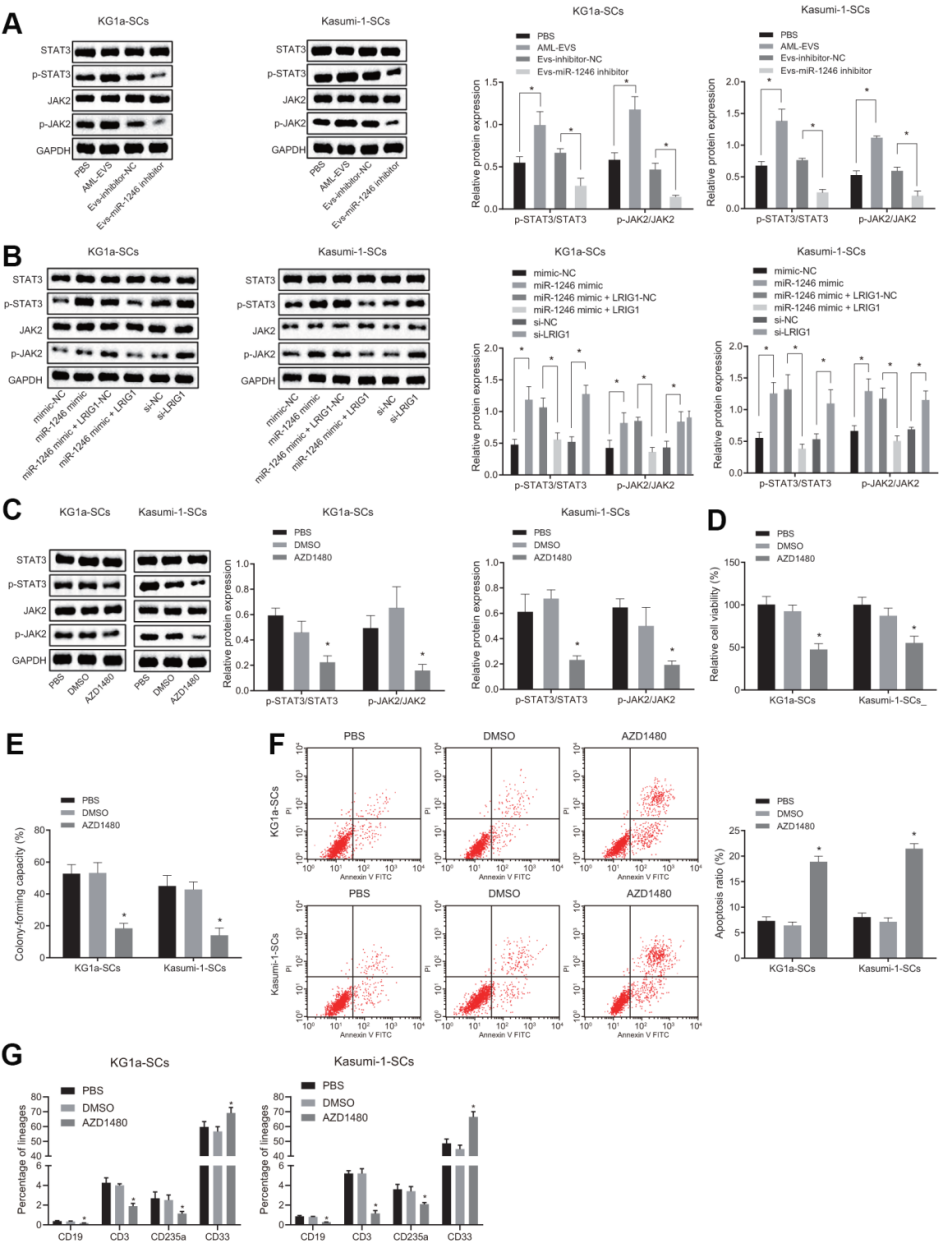
**miR-1246 targets LRIG1 and activates the STAT3 pathway, facilitating cell survival and suppressing differentiation of LSCs.** (**A**) Expression of the STAT3 pathway-related proteins (phosphorylated JAK2 and phosphorylated STAT3) in LSCs co-cultured with AML cell-derived EVs measured by western blot analysis. (**B**) Expression of STAT3 pathway-related proteins (phosphorylated JAK2 and phosphorylated STAT3) in LSCs after over-expression of miR-1246 and inhibition of LRIG1 alone or in combination demonstrated by western blot analysis. (**C**) Expression of STAT3 pathway-related proteins (phosphorylated JAK2 and phosphorylated STAT3) in LSCs after exposure to the JAK2 inhibitor AZD1480 determined by western blot analysis. (**D**) LSCs viability after exposure to the JAK2 inhibitor AZD1480 detected by MTS assay. (**E**) Colony formation of LSCs after exposure to the JAK2 inhibitor AZD1480 evaluated by colony formation assay. (**F**) LSCs apoptosis after exposure to the JAK2 inhibitor AZD1480 identified by flow cytometry. (**G**) Differentiation of LSCs after exposure to the JAK2 inhibitor AZD1480 determined by differentiation assay. * *p* < 0.05 *vs.* LSCs co-cultured with PBS, EVs-inhibitor-NC, mimic-NC, si-NC, LRIG1-NC, miR-1246 mimic + LRIG1-NC or DMSO. Data in the figures are all measurement data, expressed as mean ± standard deviation. Data among multiple groups were analyzed by one-way ANOVA. The experiments were repeated in triplicate.

LRIG1 has been reported to possess the capacity to inhibit the STAT3 pathway [16]. Inhibition of STAT3 is further associated with decreased survival rate of leukemia cells, increased apoptosis and differentiation, implying that the STAT3 pathway plays an imperative role in AML cells [22]. Thus, we speculated that miR-1246 might activate the STAT3 pathway by targeting LRIG1, thereby affecting the biological functions and differentiation of LSCs *in vitro*. As shown in Figure 6A, the expressions of STAT3 pathway-related proteins, phosphorylated JAK2 and phosphorylated STAT3, were all significantly elevated in LSCs co-cultured with AML cell-derived EVs, while being notably reduced in LSCs co-cultured with the EVs-miR-1246 inhibitor.

Furthermore, to verify whether miR-1246 activated the STAT3 pathway by targeting LRIG1, the STAT3 pathway-related protein expression patterns were evaluated after over-expression of miR-1246 and silencing LRIG1 alone or combined in LSCs. As depicted in Figure 6B, the expressions of phosphorylated JAK2 and phosphorylated STAT3 in LSCs were increased upon transfection with miR-1246 mimic, which were reversed by LRIG1 up-regulation. The transfection of si-LRIG1 enhanced the expression of phosphorylated JAK2 and phosphorylated STAT3 in LSCs, while the transfection of LRIG1 brought about the opposite results.

Subsequently, LSCs were treated with JAK2 inhibitor (AZD1480, 0.5 μM) in order to explore whether the STAT3 pathway was associated with the biological function and differentiation of LSCs *in vitro*. Firstly, the expression patterns of phosphorylated JAK2 and phosphorylated STAT3 were measured, which revealed that AZD1480 significantly down-regulated the expressions of phosphorylated JAK2 and phosphorylated STAT3 in LSCs (Figure 6C). In addition, Figure 6D, 6E illustrate that the viability and colony formation abilities of LSCs were significantly reduced, while apoptosis and differentiation were elevated after exposure to AZD1480 (Figure 6F, 6G). These results conferred that miR-1246 could indeed activate the STAT3 pathway by targeting LRIG1, thereby promoting LSCs survival and inhibiting differentiation *in vitro*.

### AML cell-derived EVs delivering miR-1246 inhibitor diminishes the tumorigenicity of LSCs *in vivo*

**Figure 7:**
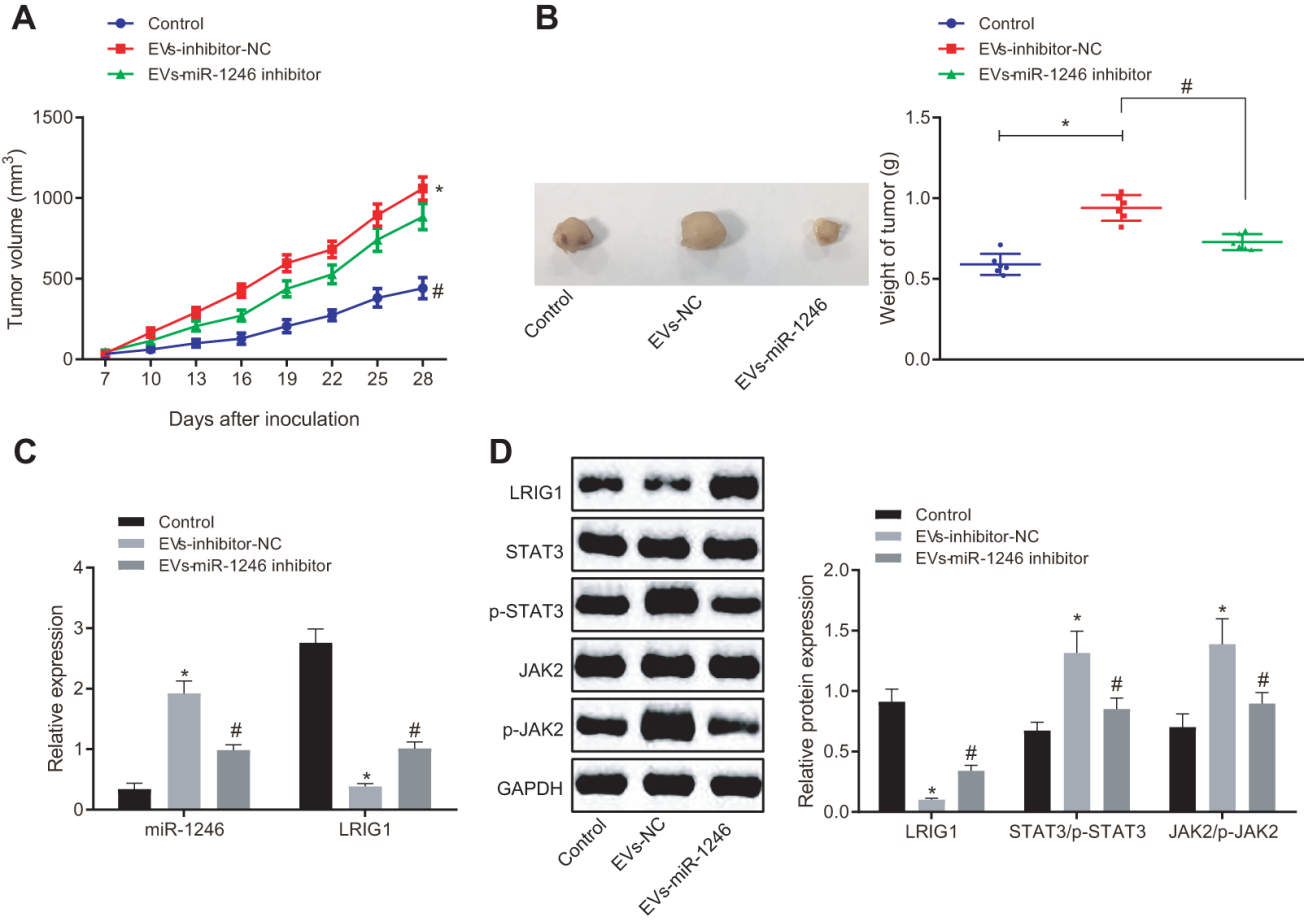
**AML cell-derived EVs deliver miR-1246 inhibitor to restrain the growth of transplantation tumors of LSCs.** Mice were subcutaneously injected with normal saline, EVs-inhibitor-NC and EVs-miR-1246 inhibitor. (**A**, **B**) Tumor formation in mice observed and recorded in real time. (**C**) Expression of miR-1246 and LRIG1 in tumor tissues of mice tested by RT-qPCR. (**D**) Expression of LRIG1 and STAT3-related proteins in tumor tissues of mice detected by western blot analysis. * *p* < 0.05 *vs.* mice injected with normal saline. #*p* < 0.05 *vs.* mice injected with EVs-inhibitor-NC. n = 6. Data in the figures are all measurement data, expressed as mean ± standard deviation. Data among multiple groups were analyzed by one-way ANOVA. Data among multiple groups at different time points were measured by repeated measurement ANOVA.

Lastly, in order to verify whether miR-1246-containing EVs from AML cells inhibited the tumor formation of LSCs *in vivo*, KG1a-SCs cells were subcutaneously injected into NOD/SCID mice to establish subcutaneously transplanted tumor models. Tumor formation in mice was observed and recorded in real time through subcutaneous injection with normal saline as NC, EVs-inhibitor-NC and EVs-miR-1246 inhibitor at fixed time points. As shown in Figure 7A, 7B, in comparison with normal saline injection, the injection of EVs-inhibitor-NC led to elevated tumor volume and weight. In contrast, the tumor volume and weight of mice were found to be significantly decreased after injection with the EVs-miR-1246 inhibitor compared to the EVs-inhibitor-NC injection. In addition, RNA analysis revealed a remarkable increase in the miR-1246 expression, while a significant decline in LRIG1 expression in mice receiving EVs-inhibitor-NC injection compared with normal saline-treated mice. Whereas, miR-1246 was observably down-regulated and LRIG1 was increased in mice injected with the EVs-miR-1246 inhibitor relative to those with EVs-inhibitor-NC (Figure 7C). Furthermore, Western blot analysis revealed that LRIG1 expression was impaired and the extents of JAK2 and STAT3 phosphorylation were enhanced as a consequence of EVs-inhibitor-NC injection in comparison with normal saline injection. Notably, opposite trends were evident in mice injected with the EVs-miR-1246 inhibitor versus EVs-inhibitor-NC (Figure 7D). These results suggested that the miR-1246 inhibitor delivered by AML cell-derived EVs repressed the tumorigenicity of LSCs *in vivo*.

## DISCUSSION

As a malignant disease of hematopoietic stem cells, AML features clonal expansion of abnormally differentiated myeloblasts [23]. EVs play a critical role in the regulation of intercellular communication and can even serve as potential vectors for delivering drugs and therapeutic genes [24]. MiRNAs-containing EVs have being developed as a new cancer diagnostic marker with complete analysis and standardized procedures [25]. The current study revealed that AML cell-derived EVs delivered miR-1246 to LSCs, thereby promoting cell viability and colony formation, and suppressing apoptosis and differentiation, which was associated with STAT3 pathway and LRIG1.

Firstly, our findings revealed that AML cell-derived EVs exhibited up-regulated levels of miR-1246. Aberrantly elevated miR-1246 expression patterns have also been documented in leukemia patients by various authors [17]. In accordance with this, miR-1246 was also over-expressed in breast cancer patients and cell lines, which further reiterates the significance of this particular miRNA in the field of oncology [26]. MiR-1246 was also highly expressed in hepatocellular carcinoma [12]. The aforementioned further reiterates the significance of this particular miR in the field of oncology. Additionally, the present findings supported that AML cell-derived EVs promoted the cell viability and colony formation while simultaneously inhibiting the apoptosis and differentiation of LSCs. EVs have been reported to be involved in gene regulation, as they function as carriers of apoptotic resistance-related protein complexes in AML [27]. Furthermore leukemia cell-derived EVs systemically suppress hematopoiesis [5] and EVs secreted by tumor cells confer potency to trafficking tumor cells which usually lack the ability to metastasize into specific organs [28].

Moreover, accumulating evidence has also highlighted the pivotal role played by LSCs in the pathogenesis and chemoresistance of AML, with high LSCs scores being correlated with poor prognoses in AML patients [29]. Authors have gone as far as to state that the lack of effective targeting and elimination modalities for LSCs is the main obstacle in the treatment of AML [18].

Tackling this head on, the findings in our study demonstrated that miR-1246-containing EVs from AML cells possess the ability to target LSCs and promote their survival. Similarly, miR-1246 has also been reported to potentially enhance the cell proliferation of leukemia cells by targeting the Notch2 pathway [17]. High expression of miR-1246 can also facilitate cell proliferation and migration of breast cancer [26]. miR-1246 plays a role in maintaining stem cell-like characteristics, as well as promoting the tumorigenesis, metastasis, angiogenesis, self-renewal and drug resistance of hepatocellular carcinoma [12].

What’s more, bioinformatics analysis in the current study revealed that LRIG1 was a target gene of miR-1246, which was further confirmed by dual luciferase reporter gene assay. Incidentally, LRIG1 is located at the chromosome band 3p14.3, the chromosomal region that is often found to be deleted in human cancers [21]. In addition, LRIG1 is known to be down-regulated in non-small cell lung cancer (NSCLC), and even highlighted to serve as a tumor suppressor in NSCLC [30]. Moreover, previous studies have also shown that LRIG1 exerts anti-oncogenic effects by inhibiting cell invasion, migration and proliferation in glioma cell microenvironment [15]. Meanwhile, miR-1246 regulating genes underlie the regulatory role of miRNAs in diseases. For instance, miR-1246 targets CCNG2 directly and functions as a tumor-promoter role in breast cancer [26]. In our study, we encountered that miR-1246 targeted LRIG1, and then activated the STAT3 pathway to promote the survival of LSCs and inhibit their differentiation. A previous study also supported the notion that inhibition of STAT3 was associated with a decrease in LSCs viability [22]. LRIG1 is also known to negatively-regulate the STAT3-dependent inflammation pathway to mediate the fate of corneal cells during repair [16].

**Figure 8:**
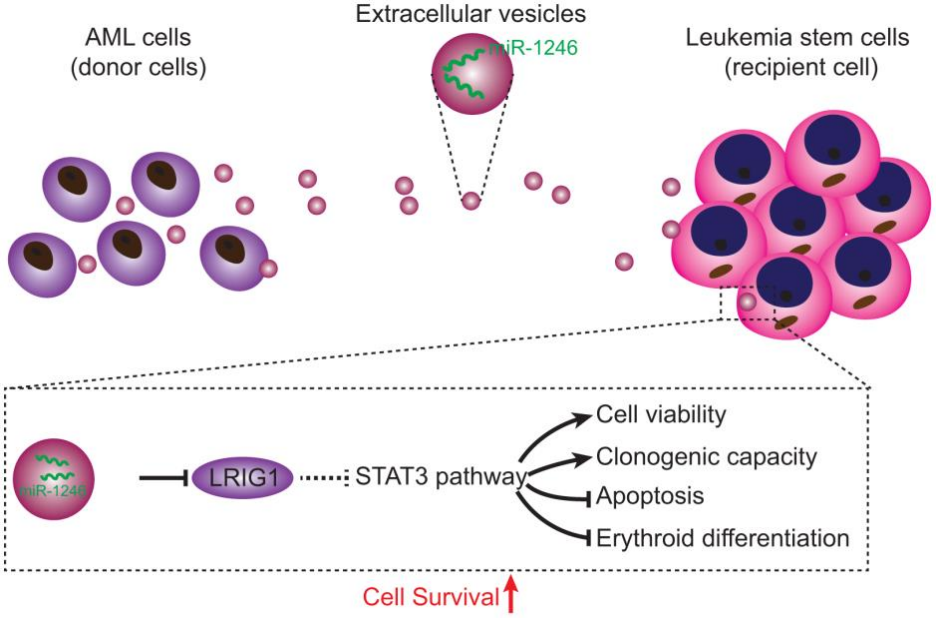
**Regulatory mechanisms of miR-1246-containing EVs from AML cells involved in LSC bioactivities in AML.** AML cells secreted EVs deliver miR-1246 to LSCs. MiR-1246 activates STAT3 pathway by targeting LRIG1, thereby promoting LSCs viability and colony formation and inhibiting cell apoptosis and differentiation, which ultimately elevates LSCs survival.

In conclusion, findings obtained in the current study demonstrated that AML cell-derived EVs carrying miR-1246 activated the STAT3 pathway by targeting LRIG1, thereby promoting LSCs viability and colony formation ability, while inhibiting cell apoptosis and differentiation, ultimately augmenting the survival of LSCs (Figure 8). Our data demonstrate the role of miR-1246-containing EVs in AML, and provides a novel and feasible approach for AML treatment. However, further studies are warranted to ascertain the effectiveness and safety of blocking the miR-1246/LRIG1/STAT3 pathway in AML.

## MATERIALS AND METHODS

### Ethics statement

All animal experiments in the current study were conducted in accordance with *the Guide for the Care and Use of Laboratory animals* published by the US National Institutes of Health and approved by the Animal Ethics Committee of Tongji Medical College, Huazhong University of Science and Technology. Extensive efforts were made to minimize the number and suffering of the included animals.

### Microarray-based gene expression profiling

Firstly, gene expression profile of the AML cell-secreted EV-mediated RNA (GSE55025) [5] was retrieved from the Gene Expression Omnibus (GEO) database (https://www.ncbi.nlm.nih.gov/geo), which contained 6 EV samples from normal cells and 6 EV samples from AML cells. The limma package of R language (http://bioconductor.org/packages/limma) was subsequently applied to screen the highly-expressed and lowly-expressed miRNAs (logFC > 3, *p* < 0.05) in AML, and then a volcano plot was drawn. Next, the R language was used to plot the expression boxplot of the miR-1246 profile. In addition, the target genes of miR-1246 were obtained using online databases including, mirDIP (http://ophid.utoronto.ca/mirDIP/), TargetscanHuma (http://www.targetscan.org/vert_71/) and miRmap (https://mirmap.ezlab.org/app/). As a result, target genes with a comprehensive score greater than 0.15 in mirDIP, with an absolute value of weighted comprehensive score greater than 0.02 in TargetscanHuma, and with a ranking of top 1000 in miRmap were screened. Online Venn diagram analysis (http://bioinformatics.psb.ugent.be/webtools/Venn/) was then carried out to obtain the intersecting target genes. The PPI analysis of intersected target genes was mapped out using the String database (https://string-db.org), with a minimum score of 0.15. PPI map was modified by the software Cytoscape (https://cytoscape.org), and genes with junction points equal to and greater than 10 (≥ 10) were screened. Next, a Venn diagram was plotted according to the screened genes and low-expressed genes related to leukemia in the TCGA database analyzed by GEPIA2. Through quantitative analysis (the detailed information can be seen in Figure 4A–4D), LRIG1 was selected for the following study. Subsequently, the binding site of LRIG1 was obtained from the TargetscanHuma. Finally, the effect network of miR-1246 in AML was analyzed.

### Cell culture and sorting

Human AML cell lines including KG1a and Kasumi-1 (ATCC, Manassas, VA, USA) were suspended in Dulbecco's Modified Eagle's Medium (DMEM) containing 100 U/mL streptomycin and penicillin, and 10% fetal bovine serum (FBS). The CD34^+^CD38^-^ fraction of KG1a and Kasumi-1 cells were defined as leukemia stem cells (LSCs). According to the manufacturer's protocol (MiltenyiBiotec, BergischGladbach, Germany), LSCs were enriched as previously described [6]. Briefly, the cell suspension was centrifuged at 300 g/min for 10 min and rinsed with phosphate buffer saline. The cell pellets were then resuspended in separation buffer and incubated with CD38-biotin. After another PBS rinse, cell pellets were resuspended in separation buffer containing anti-biotin microbeads. The filtrates (CD38^-^ cells) were collected using the LD column of the MidiMACS separator system. The CD34 MultiSort MicroBeads procedure was then applied to isolate the final CD34^+^CD38^-^ cells. During the whole sorting process, the cells were maintained at a temperature of 4° C. Finally, the sorted LSCs were analyzed using flow cytometry (PE-CD34 [550761, BD Biosciences, San Jose, USA]; FITC-CD38 [555459, BD Biosciences, San Jose, USA]). Remaining cells after LSCs isolation were defined as AML cells for further co-culture experimentation.

### Isolation and identification of EVs

After undergoing hyper centrifugation at 1 × 10^6^ g for 16 h at 4° C (Avanti J-30I, Beckman Coulter Inc., Brea, CA, USA), EVs were depleted by FBS. EV-free FBS was used to avoid the effects of EVs during following experiments. AML cell lines (KG1a and Kasumi-1) were incubated for 48-72 h. The medium was harvested, and EVs were subsequently separated by hyper centrifugation. In short, the cell culture medium was centrifuged for 10 min at 300 g, 15 min at 2,000 g, and 30 min at 12,000 g to remove the floating cells and cell debris. After passing through a 0.22 μm filter, the supernatant was hyper centrifuged again at 1 × 10^6^ g at 4° C for 2 h twice. Finally, the precipitate was resuspended with approximately 100 mL PBS for immediate use or stored at -80° C for later experimentation.

The morphology of the precipitate was examined using a transmission electron microscope (TEM). In addition, precipitate concentration and size were measured by nanoparticle tracking analysis. The isolated precipitate was diluted to a ratio of 1:10, and observed under a Nanosight NS300 nanoparticle detector (Malvern Panalytical Ltd, Malvern, UK). Precipitate particles were then dissolved in the Radio Immunoprecipitation Assay (RIPA) buffer. The protein was quantified using a bicinchoninic acid (BCA) protein analysis kit (Thermo Fisher Scientific, Rockford, IL, USA). Western blot analysis of EV was performed using the following antibodies: anti-TSG101 (ab125011, dilution ratio of 1 : 1000), anti-CD63 (ab134045, dilution ratio of 1 : 1000) and anti-beta actin (ab8227, dilution ratio of 1 : 2000) [31].

### Fluorescent labeling and EV transfer

EVs were labeled at 37° C using carboxyfluorescein succinimidyl ester (CFSE) for 30 min. Labeled EVs were then rinsed with PBS and centrifuged at 1 × 10^5^ g for 1 h to remove the excess dye [31]. CFSE-labeled EVs and LSCs were co-cultured in the culture medium for 24 h for *in vitro* analysis*.* In order to recognize the transfer of EV-containing miR-1246, Cy3 labeled miR-1246 was transfected into AML cells. Subsequently, AML cells expressing Cy3-miR-1246 were co-cultured with LSCs in a 24-well Transwell chamber for 24 h. Immunofluorescent cells were then prepared as described above. In addition, FITC Phalloidin (Yeasen Company, Shanghai, China) was applied to selectively stain the cytoskeleton of LSCs. Finally, an Olympus BX41 microscope equipped with a Charge-coupled Device (MagnaFire, Olympus, Beckman Coulter Inc., Brea, CA, USA) was used to observe the EVs.

### Cell culture and transfection

Cultured AML cells were transfected with inhibitor-NC, miR-1246 inhibitor, mimic-NC and miR-1246 mimic. Cultured LSCs were subsequently transfected with mimic-NC, miR-1246 mimic, si-NC, si-LRIG1, DMSO (0.5 μM, Sigma-Aldrich Chemical Company, St Louis, MO, USA), AZD1480 (JAK2 inhibitor, 0.5 μM, Selleckchem Chemicals, Houston, TX, USA) and co-transfected with miR-1246 mimic + LRIG1-NC, miR-1246 mimic + LRIG1 [32]. First, AML cells were transfected with miR-1246 mimic or inhibitor, and then EVs were extracted from the AML cells and incubated with recipient LSCs. These cells were seeded in 12-well plates with DMEM medium for 24 h prior to transfection. Upon reaching 70% confluence the cells were resuspended in the 12-well plate with serum-free DMEM medium, and transfected accordingly. After transfection, the cells were incubated in 5% CO_2_ at 37° C for 6-8 h. Subsequently, culture medium was replaced with FBS containing medium, and the cells were incubated for 24 - 48 h for the follow-up experiments. Transfected AML cells were used for EV isolation. The cells were treated with 75 nM miR-1246 inhibitor, 100 nM miR-1246 mimic, 70 nM si-LRIG1, 100 nM over-expressed LRIG1, and their relative negative control (NC) combined or separately. All the aforementioned plasmids were supplied by Guangzhou RiboBio Co., Ltd (Guangzhou, China).

### Cell viability assay

LSCs were seeded in 96-well plates (100 μL/well) and cultured until reaching the logarithmic phase of growth. After 24 h of incubation, the cells were transfected with 3 parallel wells in each group. Then, 3-(4,5-dimethylthiazol-2-yl)-5-(3-carboxymethoxyphenyl)-2-(4-sulfophenyl)-2H-tetrazolium (MTS) cell viability and cytotoxicity assay kits (BB-4203-3, Shanghai BestBio Co., Ltd, Shanghai, China) was applied to detect the viability of LSCs. Briefly, after 48 h of transfection, 10 μL of MTS solution was added to each well and the cells were incubated for 1- 4 h at 37° C. The absorbance value of each well at a wavelength of 490 nm was detected using a multifunctional microplate reader. Blank wells (i.e. medium and MTS solution without cells), control wells (i.e. cells only) and 3 parallel wells were set for each group. Cell viability was calculated using the following formula: Cell viability = (optical density (OD) of experimental cells–OD in blank well)/(OD in control well –OD in blank well) × 100%.

### Colony formation assay

EVs (collected from 6 × 10^7^-7 × 10^7^ AML cells) were co-cultured with 1 × 10^6^ LSCs for 48 h, with PBS serving as the NC. Human Methylcellulose Complete Media (R&D Systems, Inc, Minneapolis, MN, USA) were used for colony-forming unit analysis. Briefly, 5000 LSCs were added into a 35 mm dish. Cells were then cultured in 5% CO_2_ at 37° C with more than 95% humidity for 7 days. On day 7, an optical microscope was employed to observe and count the formed colonies [5].

### Differentiation assay

In the differentiation experiment, LSCs were cultured with Iscove’s Modified Dulbecco’s Medium (IMDM) medium (Gibco, Carlsbad, CA, USA) containing 10% FBS, 100 ng/mL recombinant human stem cell factor (rhSCF), 100 ng/mL rhFlt3 and 100 ng/mL for recombinant human thrombopoietin (rhTPO) with 5% CO_2_ at 37° C for 24 h. After culture, LSCs were treated with 1 μg/mL AML cell-derived EVs for 7 days. Next, LSCs were collected and labeled with CD19-PE (555413, BD Biosciences, San Jose, CA, USA), CD33-APC (561817, BD Biosciences, San Jose, CA, USA), CD3-APC (561811, BD Biosciences, San Jose, CA, USA), and CD235a-FITC (559943, BD Biosciences, San Jose, CA, USA) for 30 min. Cells were then detected using a FACS Calibur (BD Biosciences, San Jose, CA, USA) and analyzed with the Cell Quest software (BD Biosciences, San Jose, CA, USA) [33].

### Apoptosis analysis

Apoptosis analyses were performed using Annexin V and Propidium iodide kits (Thermo Fisher Scientific, Rockford, IL, USA). Briefly, LSCs were collected and rinsed with PBS and binding buffer. After rinsing, 5 μL of Annexin V were added to the cells and incubated for 15 minutes without light exposure. After another rinse, LSCs were treated with then binding buffer and 5 μL of propidium iodine. Lastly, the cell mixtures were incubated on ice at 2–8° C and analyzed by flow cytometry using a FACSAria II Special Order System (BD Biosciences, San Jose, CA, USA).

### Reverse transcription quantitative polymerase chain reaction (RT-qPCR)

Total RNA content was extracted from cells or EVs using a miRNeasy or RNeasy kits (Qiagen Inc, Hilden, Germany) and quantified using a NanoDrop^TM^ 2000 spectrophotometer (Thermo Fisher Scientific, Rockford, IL, USA). Next, the obtained RNA was reverse transcribed into complementary DNA (cDNA) following the instructions of SuperScript III First-Strand Synthesis kit (Invitrogen, Carlsbad, CA, USA) with oligo (dT) primers. RT-qPCR was then performed with the SYBR Green PCR kit (Applied Biosystems, Carlsbad, California, USA) using an ABI PRISM 7500 real-time PCR system (Omega Bio-tek Inc, Norcross, GA, USA). The primer sequences are shown in the Table 1. All aforementioned primer sequences were synthesized by Guangzhou RiboBio Co., Ltd (Guangzhou, China). The relative expression of genes was calculated by means of relative quantification (2^-∆∆Ct^ method) with glyceraldehyde-3-phosphate dehydrogenase (GAPDH) serving as the internal control [34]. For miRNA quantification, TaqMan assay kits (Applied Biosystems, Carlsbad, California, USA) were applied for reverse transcription and RT-qPCR, with U6 snRNA serving as the endogenous control [5].

**Table 1:** Primer sequences for RT-qPCR.

**Gene**	**Primer sequence (5'-3')**
miR-1246	F: TGAAGTAGGACTGGGCAGAGA
	R: TGTTTGCAATAGCCCTTTGAG
U6	F: CTCGCTTCGGCAGCACATA
	R: GTGCAGGGTCCGAGCT
LRIG1	F: GGTGAGCCTGGCCTTATGTGAATA
	R: CACCACCATCCTGCACCTCC
GAPDH	F: TGCACCACCAACTGCTTAGC
	R: GGCATGGACTGTGGTCATGAG

Note: RT-qPCR, reverse transcription-quantitative polymerase chain reaction; F, forward; R, reverse; miR-1246, microRNA-1246; LRIG1, leucine rich repeats and immunoglobulin like domains 1; GAPDH, glyceraldehyde-3-phosphate dehydrogenase.

### Western blot analysis

Total protein content was extracted from tissues or cells using an enhanced RIPA lysis buffer (R0010, Beijing Solarbio Science and Technology Co., Ltd, Beijing, China) in accordance with the manufacturer’s instructions. After undergoing lysing at 4° C for 15 min, the cells were centrifuged at 15,000 g for 15 min and quantified using a BCA protein assay kit (Shanghai Yeasen Co., Ltd., Shanghai, China) after collecting the supernatant. The proteins were then separated using polyacrylamide gel electrophoresis, and then transferred onto a polyvinylidene fluoride membrane using the wet-transfer method. After being blocked with 5% BSA at room temperature for 1 h, the membrane was incubated at 4° C overnight with primary rabbit antibodies (Cell Signaling Technology, Beverly, MA, USA) to LRIG1 (dilution ratio of 1: 1000, 12752), phosphorylated STAT (dilution ratio of 1: 2000,9145), total STAT3 (dilution ratio of 1: 1000, 8768), phosphorylated JAK2 (dilution ratio of 1:1000, 3771), total JAK2 (dilution ratio of 1:1000, 3230), with GAPDH (dilution ratio of 1 : 1000, 5174) serving as the control. After 3 rinses (5 min/time) with Tris-buffered saline with Tween (TBST), the membrane was incubated with horseradish peroxidase (HRP)-labeled secondary antibody goat anti-rabbit IgG (dilution ratio of 1 : 2000, 7074) for 1 h. The membrane was then rinsed with TBST thrice (5 min/time), and developed with the developing solution. The quantitative analysis of protein was performed using the ImageJ 1.48u software (National Institutes of Health, Bethesda, Maryland, USA). The GAPDH expression was regarded as the endogenous control for relative protein expression.

### Dual luciferase reporter gene assay

The artificially synthesized LRIG1 3'UTR gene fragment was inserted into the psiCHECK-2 vector (Promega Corp., Madison, WI, USA), and the complementary sequence mutation site of sub-sequence was designed based on the wild type sequence of LRIG1, which was then inserted into the reporter plasmid of the psiCHECK-2 vector. The correctly sequenced luciferase reporter plasmids wild type-LRIG1-3'-UTR (LRIG1-3'-UTR^W^, 100 ng) and mutant type-LRIG1-3'-UTR (LRIG1-3'-UTR^M^, 100 ng), were co-transfected into 4 × 10^5^ HEK-293T cells (CRL-1415, Shanghai Xinyu Biological Technology co., Ltd, Shanghai, China) with miR-1246 mimic or mimic-NC (2 nM, Dharmacon, Inc, Chicago, IL, USA), respectively. The cells were collected and lysed 48h after transfection. A luciferase assay kit (RG005, Beyotime Biotechnology Co., Shanghai, China) was then applied to detect luciferase activity using a Glomax20/20 luminometer (Promega Corp., Madison, WI, USA) [5].

### Tumor formation in mice

Nonobese diabetic/severe combined immunodeficient (NOD/SCID) mice (aged 6-9 weeks old) were procured from the Beijing Vital River Laboratory Animal Technology Co., Ltd. (Beijing, China). A total of 0.5 × 10^5^ LSCs were injected subcutaneously into both flanks of the mice. Subsequently, EVs from AML cells transfected with miR-1246 inhibitor were extracted and injected subcutaneously into the mice. The mice were then injected with EVs-inhibitor-NC, EVs-miR-1246 inhibitor, or their relative NC combined or separately. Tumor volume was observed and calculated in real-time according to the following formula: V (tumor volume) = A (the long axis) × B (the short axis)^2^/2(mm^3^). When tumor volume reached 1,000 mm^3^, all mice were euthanized and tumors were weighted. Total RNA and protein contents were extracted from the tumor tissues to detect the expression patterns of miR-1246/LRIG1/STAT3-related pathway in each group.

### Statistical analysis

All experiments were performed in triplicate. Statistical analyses were performed using the SPSS 21.0 statistical software (IBM Corp., Armonk, NY,

USA). Measurement data were presented as mean ± standard deviation. Paired *t*-test was applied for comparisons between two groups. Data among multiple groups were analyzed by one-way analysis of variance (ANOVA). Data among multiple groups at different time points were analyzed by repeated measurement ANOVA. A value of *p*< 0.05 was considered statistically significant.

## Supplementary Material

Supplementary Table 1
